# Impact of three co-occurring physical ecosystem engineers on soil Collembola communities

**DOI:** 10.1007/s00442-022-05152-5

**Published:** 2022-04-07

**Authors:** D. D. G. Lagendijk, D. Cueva-Arias, A. R. Van Oosten, M. P. Berg

**Affiliations:** 1 Section Animal Ecology, Department of Ecological Science, Vrije Universiteit Amsterdam, De Boelelaan 1085, 1081 HV Amsterdam, The Netherlands; 2 School of Life Sciences, University of KwaZulu-Natal, Private Bag X01, Scottsville, 3209 South Africa; 3 Conservation and Community Ecology Group, Groningen Institute for Evolutionary Life Sciences, University of Groningen, P.O. Box 11103, 9700 CC Groningen, The Netherlands

**Keywords:** Soil fauna, Grazing, Non-trophic interactions, Bioturbation, Vegetation structure

## Abstract

The interplay between organisms with their abiotic environment may have profound effects within ecological networks, but are still poorly understood. Soil physical ecosystem engineers (EEs) modify the abiotic environment, thereby potentially affecting the distribution of other species, such as microarthropods. We focus on three co-occurring physical EEs (i.e. cattle, vegetation, macrodetritivore) known for their profound effect on soil properties (e.g. pore volume, microclimate, litter thickness). We determined their effects on Collembola community composition and life-form strategy (a proxy for vertical distribution in soil) in a European salt marsh. Soil cores were collected in grazed (compacted soil, under short and tall vegetation) and non-grazed areas (decompacted soil, under short and tall vegetation), their pore structure analysed using X-ray computed tomography, after which Collembola were extracted. Collembola species richness was lower in grazed sites, but abundances were not affected by soil compaction or vegetation height. Community composition differed between ungrazed sites with short vegetation and the other treatments, due to a greater dominance of epigeic Collembola and lower abundance of euedaphic species in this treatment. We found that the three co-occurring EEs and their interactions modify the physical environment of soil fauna, particularly through changes in soil porosity and availability of litter. This alters the relative abundance of Collembola life-forms, and thus the community composition within the soil. As Collembola are known to play a crucial role in decomposition processes, these compositional changes in litter and soil layers are expected to affect ecosystem processes and functioning.

**Supplementary Information:**

The online version contains supplementary material available at 10.1007/s00442-022-05152-5.

## Introduction

Ecological networks consist of various types of interactions among (a)biotic components. One of these interactions is the interplay between organisms with their (non-resource) abiotic environment. An example of such organisms are physical ecosystem engineers (hereafter EEs), as a term first used by Jones et al. (1994). By modifying their physical environment EEs have a profound effect on ecosystem functioning and biodiversity (Jones et al. 1997; Losapio et al. 2021). In many ecosystems multiple engineers with different modifying actions co-occur, interact and together shape the structure of ecosystems, which can have repercussions for non-engineering organisms (see among others Eldridge et al. 2010; Jones 2012). However, not much is known about how multiple EEs impact other species, particularly in soils.

Physical EEs modulate their (a)biotic environment, through for example destructive feeding, building structures or burrowing activities (Kerley et al. 2008; Baker et al. 2013; Coggan et al. 2018). Well-known terrestrial examples of EEs are elephant, prairie dogs, termites and earthworms (Wright and Jones 2006; Lavelle et al. 2006; Pringle 2008; Baker et al. 2013; Lagendijk et al. 2016). They induce a structurally mediated (a)biotic state change thereby (in)directly affecting resource availability and habitat for other organisms (Jones et al. 1994, 1997). Therefore, these often long-lasting environmental modulations can affect community dynamics of non-engineering species, as these changes may favour some species over others (Jones et al. 1997). In addition to structural effects, engineering also affects ecosystem processes, such as litter degradation, sediment retention, primary production and soil formation and structure, which effects may also ultimately cascade onto other taxa (Orwin et al. 2016; Ferlian et al. 2018; De Almeida et al. 2020). However, little is known of these effects on soil fauna.

Here we investigated the combined effect which three co-occurring physical EEs exert on Collembola communities of a Dutch salt marsh. Collembola, an abundant non-engineering group of microarthropods, contribute to ecosystem processes by playing a key part in nutrient cycling through their facilitating role in decomposition (Rusek 1998). In the studied salt marsh, cattle are the primary EE. They compact the soil through trampling, reduce soil porosity and connectivity, and increase anoxic conditions (Schrama et al. 2013; Howison et al. 2017; Keshta et al. 2020), all reducing habitat space for Collembola. Furthermore, defoliation through grazing results in mosaics with short and tall vegetation patches (i.e. palatable vs. unpalatable plant species) (Bakker et al. 1984; Kotanen and Abraham 2006). Under short vegetation available resources for litter-inhabiting Collembola are low, due to a reduction in the amount of litter. Plants are the secondary EE. Tall plants provide shade, influencing soil microclimate conditions (Willott 1997; Angers and Caron 1998), and produce more litter, thereby creating habitat for Collembola (Fujii et al. 2020). Plants also increase pore space through root growth and shoots which push the soil upwards when emerging in spring (Howison et al. 2017), an impact on soil structure they share with another secondary EE, the bioturbating amphipod *Orchestia gammarellus.* Through digging of burrows, thereby mixing dead organic matter with mineral soil and increasing pore diameter, pore connectivity, soil moisture levels and soil aeration (Wilkinson et al. 2009; Howison et al. 2015), *O. gammarellus* potentially creates favourable conditions and habitat for Collembola (Eaton et al. 2004; De Almeida et al. 2020). This bioturbator is generally absent from grazed salt marshes responding to soil compaction by grazers (Schrama et al. 2013), while the increase in light levels associated with short vegetation negatively affects their presence (Thakur et al. 2014).

We determined the effect of soil compaction (proxy for trampling by large grazers) and vegetation height (proxy for defoliation and decompaction by plants and amphipods in the absence of cattle) on soil properties and Collembola community composition and life-form within the soil. We expected the number of pores, mean pore diameter and air-filled porosity to be reduced in the presence of grazers (due to soil compaction), but to be positively influenced by the presence of *O. gammarellus*, especially in tall vegetation without grazing*.* As soil invertebrates are dependent on habitable pore space (Larsen et al. 2004), we expected lower Collembola abundance and species richness with soil compaction due to low soil porosity, irrespective of vegetation height. Consequently, we expected different Collembola community compositions across the different compaction and vegetation height treatments. In tall vegetation plots with grazing we predicted a higher relative abundance and species richness in litter compared with soil due to low soil porosity. Moreover, in compacted soil we anticipated a relatively low abundance of hemiedaphic and especially euedaphic (deep soil-dwelling) Collembola due to smaller pore space compared with decompacted soils with tall vegetation due to greater soil porosity. To determine these effects, we used a full-factorial experiment of compaction (using exclosures) and vegetation height (short vs. tall).

## Materials and methods

### Study area

This study was conducted on Schiermonnikoog (53°30' N, 6°10' E), a barrier island in the Dutch Wadden Sea. Schiermonnikoog shows a 10 km long chronosequence in natural salt marsh development, with the oldest marsh over 220 years of age (see Olff et al. 1997 for a detailed description of the chronosequence). These salt marshes were grazed by cattle till 1958 and again from 1972 onwards (Bakker 1985). Nowadays, seasonal grazing takes place from May to November with a maximum cattle density of 0.5 animal ha^−1^ (Bos et al. 2002). Other grazing species utilising the marsh are hares (*Lepus europaeus*) and geese (e.g. *Branta bernicla*, *B. leucopsis*) (Kuijper et al. 2004). The vegetation of the western and most late successional salt marsh (our study area) is dominated by the tall grass sea cough (*Elytrigia atherica)*. Mean temperature is 8.6 °C and annual rainfall is 806 mm (www.en.climate-data.org). The soil consists of sand with a 16 cm thick layer of silt of marine origin (Schrama et al. 2012).

### Experimental design

We used four existing cattle exclosures on the 180 years old part of the salt marsh chronosequence. These exclosures were set up in 1973 to study the interaction between cattle grazing and sea cough dominance in a grazed part of the salt marsh. The four exclosure sites were located between 117 and 150 m + NAP (Amsterdam Ordnance datum), and were relatively safe from flooding by sea water (winter: 6-2 inundations per month; summer: 2-0 inundations per month following Howison et al. 2015). In 1973, the exclosures (ca. 50 × 12 m) were placed on a grazed part of the salt marsh, with short vegetation and compacted soil, very much alike the current situation (Bakker 2014). The fence of the exclosures was electrified during the grazing season, representing a treatment of no grazing and thus no soil compaction, as outside the exclosure the soil was strongly compacted due to trampling by grazing cattle (Online Resource 1). The almost 50 years of excluding grazers, hence trampling, from the exclosures resulted in tall vegetation with decompacted soil due to the activity of roots and shoots, and bioturbating *O. gammarellus* (Schrama et al. 2013). In addition, within each level of compaction there were two levels of vegetation height (short vs. tall), in an orthogonal design. In the exclosures, a 3 × 3 m of the enclosed area was hand-mown twice a year using a brushcutter, in early and late summer (Schrama et al. 2012) to mimic cattle grazing, avoiding trampling of the area during mowing. This resulted in short and tall vegetation patches protected from cattle trampling. The vegetation outside the exclosures consisted of a natural short-tall vegetation mosaic, where short-grazed (few cm height) multi-species vegetation was interspersed with patches of species-poor tall vegetation (approximately 30 cm height) dominated by the unpalatable sea rush (*Juncus maritimus)* mixed with sea couch (patch size 10–300 m^2^: Howison et al. 2015). This tall vegetation was not grazed, but the soil was compacted due to trampling (Online Resource 1). Tall vegetation compositions in and outside the exclosure are the same, while the short vegetation compositions of grazed and mown plots were very similar (following species abundances in Chen 2020). The mean vegetation height of the low and tall vegetation patches was somewhat lower outside than inside the exclosures (see “Results”). This is because cattle pull off the grass closer to the ground than the hand-mower can cut the grass. In addition, cattle still walk through the tall vegetation patches making the tall grass partly ‘fall over’ by which the vegetation becomes structurally of shorter stature in the presence of cattle, while the plant leaves remain of similar length (and biomass) to the ones within the exclosures (pers. obs. MPB). The exclosure remained accessible to hares and geese at all times.

This experimental set-up resulted in four different treatments each with four replicates, two each per elevation: (1) compacted soil, short-grazed vegetation (GS), (2) compacted soil, tall vegetation (GT), (3) decompacted soil, short-mown vegetation (US) and (4) decompacted soil, tall vegetation (UT). Unfortunately, one of the exclosures did not include a mowed treatment; the other three sites did indeed include all four treatments.

### Data collection

#### Vegetation height and litter layer height

Vegetation height was measured in April and May 2017. Five random measurements to the nearest 0.5 cm were taken in each treatment, using a Styrofoam drop disc following Van Klink et al. (2016). The height of the litter layer was measured five times per plot to the nearest mm in May 2017.

#### Macrodetritivores: *Orchestia gammarellus*

The presence of the macrodetritivore *O. gammarellus* was quantified in March 2017, April 2017 and March 2019. The latter sampling period was included to illustrate similar patterns across years. In each plot, individuals were hand-sorted and counted along 1 m × 10 cm strip transects; two replicates per plot in 2017 and three replicates per plot in 2019. An individual search takes 5–20 min depending on vegetation height and structure.

#### Soil properties using X-ray tomography

Three soil cores (10 cm Ø, 8 cm height) were collected per plot in April 2017, each spaced at least one m apart within plots, resulting in a total of 45 cores. Samples were stored in closed plastic buckets (11 cm Ø, 10 cm height), transported to the Shared Research Facilities at Wageningen University & Research in The Netherlands, and cooled at 4 °C. The 3D structure of the soil was analysed using the Phoenix v[tome]x m (Waygate Technologies), which is an X-ray computed tomography scanner (XRT-CT). The settings of the X-ray source were 200 kV and 150 µA. This will not affect Collembola. The spatial resolution was 79.4 μm. During a full 360° rotation, 1500 X-ray projections were made (retrieved from three averaged images per projection with an exposure time of 250 ms each). The total acquisition time was approximately 25 min. Datosx was used to reconstruct the individual projections into a 3D matrix (image). This image was processed and analysed using AVIZO 9 software (Thermo Fisher Scientific). During the analysis, a cylinder with dimensions of 8 × 6 cm (diameter × height) was taken from the sample to exclude potential edge effects from the circumference of the core. Pore space volume, and number and diameter of pores were extracted from Avizo. Air-filled porosity (%) was calculated dividing pore volume by soil volume.

#### Collembola

After XRT-analysis, the intact soil cores were transported to the Vrije Universiteit Amsterdam and placed in Tullgren funnels for extraction of soil fauna following Van Straalen and Rijninks (1982). Collembola were preserved in 70% ethanol and identified to species using Hopkin (2007) and Fjellberg (1998, 2007).

#### Collembola life-form

We allocated Collembola species to three life-form categories reflecting their vertical distribution within the soil column (following Gisin 1943 and Salmon et al. 2014): epigeic (surface-living), hemiedaphic (sub-surface-living) and euedaphic (soil-dwelling) species. These life-form categories were derived from a set of morphological traits (e.g. level of pigmentation, number of ocelli, size of furca) of which shared values of these traits culminate into one of these three life-forms. Life-forms defined by this trait syndrome reflect an adaptation to soil life and correspond with the vertical position of species within the soil. For example, euedaphic species which are deep-soil dwellers are white, blind and have no or a strongly reduced furca, while epigeic species are colourful, have the maximum of eight ocelli and a long furca.

The presence of litter might affect the impact of soil compaction on Collembola as it acts as additional habitat (Rusek 1998; Fujii et al. 2020). To determine the community composition of Collembola in litter and corresponding soil, twelve additional soil cores to the 45 cores already scanned were collected in 2017. Cores were collected in tall vegetation only, as the amount of litter was absent or low in short vegetation patches (see “Results”). This resulted in six cores with compacted soil (i.e. grazed) and six cores with decompacted soil (i.e. ungrazed), one from each of the four replicate plots and two additional cores to increase the number of samples. The fresh litter layer was separated from the soil column immediately after collection. Within the soil column, a thin layer of buried litter mixed with sediment may be present. During each inundation event litter is covered by a thin layer of sediment, on which the new litter again accumulates. As a result, three layers can be present; a fresh litter layer on top of the soil and within the soil a few cm thin layer of litter mixed with sediment on top of a solid clay layer. This was most pronounced within ungrazed areas (with tall vegetation) as most litter was available here (pers. obs. DDGL, MPB; Meyer et al. 1995; Online Resource 1). Collembola were extracted from both the fresh litter layer and the soil, and stored as described above. Given the short time interval after the first collection date we did not expect differences in the level of soil compaction, hence no 3D structural analyses were performed on these cores.

### Statistical analyses

#### Vegetation and litter layer thickness, Orchestia gammarellus abundance and soil properties

All analyses were performed in R 3.6.3 (R Core Team 2020). To determine the effect of soil compaction (i.e. grazing vs. no grazing) and vegetation tallness (i.e. short vs. tall) on vegetation height, litter layer thickness and soil properties we used mixed linear effects models (lmerTest package: Kuznetsova et al. 2017) with a Gaussian distribution, using AICc backward model selection. AICc was used instead of AIC due to relatively small sample sizes. Soil compaction and vegetation height were included as fixed factors as well as their interaction, unless otherwise stated. Site was included as a random factor. To determine differences among the four treatments the resulting model was run again with treatment as a fixed factor, followed by a Tukey post hoc test (emmeans package; Lenth 2020).

Data of vegetation height and litter layer thickness were log-transformed prior to analyses. In the analyses of vegetation height, we added sampling period as a random factor to the models and omitted the interaction factor between compaction and vegetation height. We used Mann–Whitney *U* tests to determine an effect of compaction and vegetation height on *O. gammarellus* abundances as these data were not normally distributed. Pairwise comparisons among treatments were assessed using a non-parametric post hoc test (dunn.test package: Dinno 2017). Air-filled porosity data were arcsine transformed after converting the percentage data into proportions. Number of pores were log-transformed prior analysis.

#### Collembola community composition

Collembola species richness was calculated as the number of species present per core. Abundance was calculated as the sum of all Collembola specimens per core. The effect of soil compaction and vegetation height (incl. their interaction) on Collembola species richness and abundance were tested using a two-way ANOVA, after log-transformation of the data. The effect of soil compaction and vegetation height (incl. interaction) on Collembola community composition were analysed using PERMANOVA (vegan package: Oksanen et al. 2019), based on 9999 permutations. Species communities across treatments were visualised in a NMDS ordination using Bray–Curtis dissimilarity and a maximum of 9999 iterations. We ran a similarity percentage analysis (SIMPER) to identify which species contributed most to differences among the four treatments.

#### Life-form distribution of Collembola

The effect of soil compaction and vegetation height on Collembola life-form distribution was determined using community weighted means (CWM) of life-forms. We allocated a trait score of 1, 2 and 3 to euedaphic, hemiedaphic and epigeic species respectively, following Bokhorst et al. (2017). For each sample the CWM of life-forms was calculated by multiplying the respective scores with the relative abundance of each species, which was then summed per sample. Lower values thus represent dominance of euedaphics (deep-soil dwelling) species and conversely, high values indicate dominance of epigeic species in the community. The effect of soil compaction and vegetation height (incl. their interaction) on CWM of life-forms was analysed using a two-way ANOVA.

Subsequently, we determined the effect of soil compaction, vegetation height and their interaction on species richness, abundance and species composition for epigeic, hemiedaphic and euedaphic species groups separately. Species richness was analysed using Mann–Whitney *U* tests. Abundance was analysed using two-way ANOVAs after log-transformation of the data, with the exception of the abundance data of hemiedaphics which were analysed using a Mann–Whitney *U* test. Species composition per life-form was analysed using PERMANOVA as described above. Species composition across stratum and compaction were analysed similarly.

## Results

### Vegetation height and litter layer thickness

**Fig. 1:**
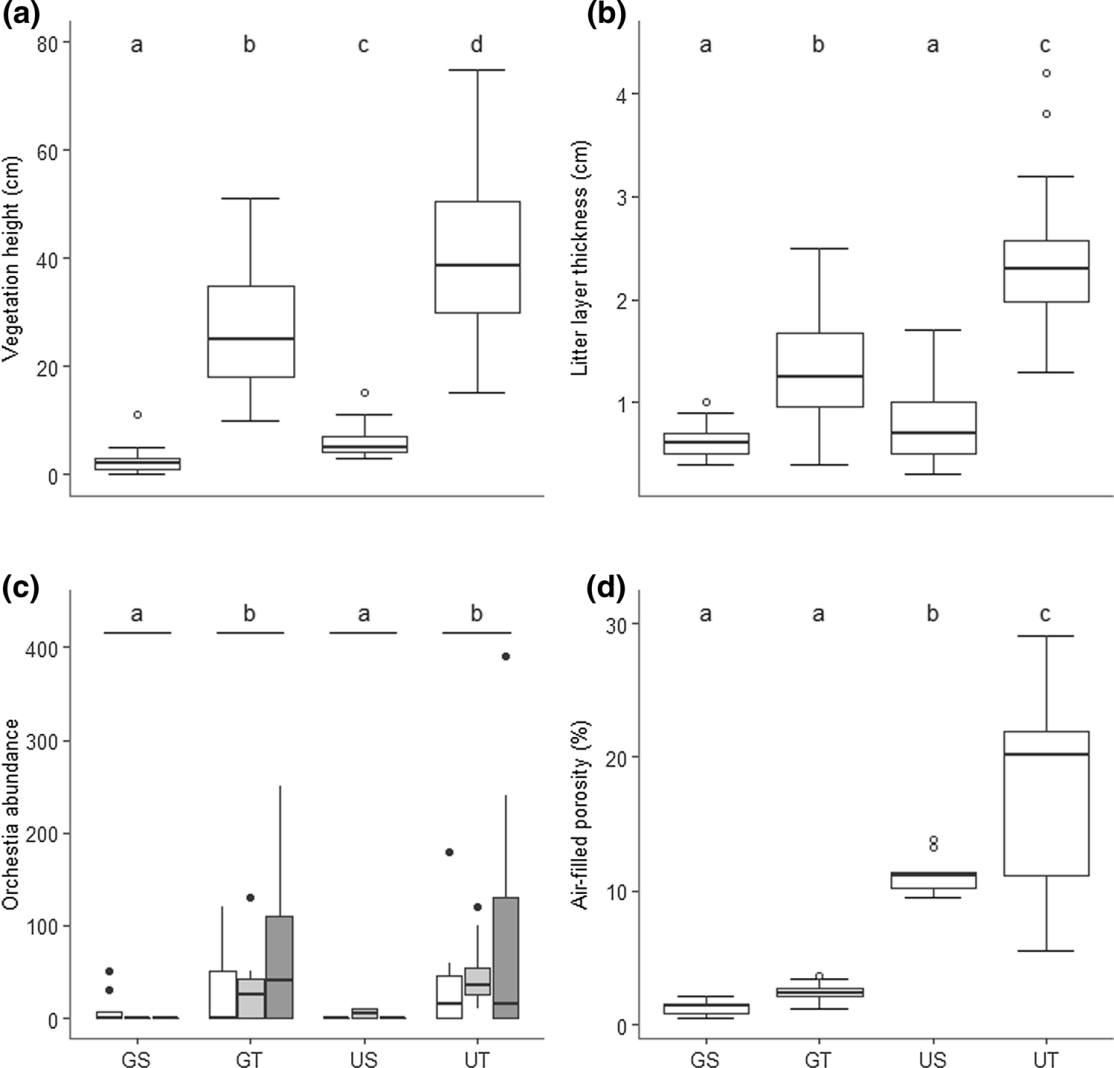
The effect of grazing large herbivores and defoliation/mowing on **a** vegetation height, **b** thickness of the litter layer, **c** *Orchestia gammarellus* abundance and **d** air-filled porosity as a proxy for compaction. Boxplots show range (whiskers), 25 and 75% quartiles (box), median (thick line) and outliers (circles). Letters within each subfigure indicate significant differences among treatments. Treatments: *GS* grazing, short vegetation, *GT* grazing, tall vegetation, *US* no grazing, short vegetation, *UT* no grazing, tall vegetation

The exclusion of grazing resulted in a tall vegetation with a mean height of 40.2 ± 14.6 cm (mean ± SD; Fig. 1a; Table 1; Online Resource 2). Mowing in the exclosures reduced this vegetation height to a mean of 5.9 ± 2.5 cm. That was not as short as in the presence of grazers outside the exclosures, where mean vegetation height was only 2.3 ± 1.8 cm. Even in the presence of grazers we observed patches of tall vegetation, due to the presence of *J. maritimus* which protects surrounding plants from herbivory, resulting in a mean vegetation height of 27.1 ± 11.5 cm. Tall vegetation height was shorter with grazing than without grazing (Table 1; Fig. 1a; Online Resource 2).

**Table 1:** Significant effects of compaction and vegetation height on physical ecosystem engineering parameters and Collembola

Parameter	Compaction		Vegetation height		Compaction × vegetation height		Model	
	Test statistic	*P*	Test statistic	*P*	Test statistic	*P*	*Χ*^*2*^	*P*
Vegetation height	*t* = − 8.60	< 0.0001	*t* = 30.61	< 0.0001			309.60	< 0.0001
Litter layer thickness			*t* = 11.04	< 0.0001	*t* = − 4.13	0.0001	94.90	< 0.0001
*Orchestia* abundance			*W* = 554.50	< 0.0001				
Air-filled porosity	*t* = − 10.09	< 0.0001	*t* = 4.98	< 0.0001	*t* = − 2.20	< 0.05	96.70	< 0.0001
Number of soil pores	*t* = − 8.95	< 0.0001	*t* = 4.25	< 0.001			54.00	< 0.0001
Species richness	*F* = 13.12	< 0.001	*F* = 6.62	< 0.05		0.06^a^		

^a^Marginally significant result reported, but not included in the final model

The thickness of the litter layer did not differ within short vegetation between grazed and ungrazed sites, but was greater in tall vegetation, particularly in ungrazed sites (Table 1; Fig. 1b; Online Resource 2).

### Soil compaction

Air-filled porosity, an indicator of soil compaction, was affected by both grazer presence and vegetation height. With grazers, air-filled porosity was similar between short (1.29 ± 0.53; mean ± SD) and tall vegetation (2.39 ± 0.76), but porosity was greater in the exclosures, particularly under tall vegetation (17.64 ± 7.36 vs. 11.19 ± 1.49 under short vegetation) (Table 1; Fig. 1d; Online Resource 2). There were fewer pores present with grazers present, but more under tall vegetation (Table 1; Online Resource 2, 3, 4).

The pore space size distributions showed an approximate inverse J-shaped curve in all treatments, indicating many small pore spaces and fewer large pore spaces (Online Resource 4). The grazed treatments had relatively few pore spaces larger than 4.5 mm^3^ [short vegetation: 0.69 ± 0.14 (mean pore space ± SD); tall vegetation: 0.78 ± 0.16], while these were present in the ungrazed treatments (short vegetation: 1.08 ± 0.16; tall vegetation: 1.16 ± 0.32). However, the largest pore spaces were present in the ungrazed treatments with tall vegetation (Online Resource 4).

*Orchestia gammarellus* abundance was greater under tall vegetation, with or without grazers present (mean ± SD; grazing: tall vegetation 45.71 ± 61.31 vs. short vegetation 2.86 ± 10.84; no grazing: tall vegetation 60.36 ± 91.31 vs. short vegetation 1.43 ± 3.59), and showed consistent patterns over the three sampling periods (Table 1; Fig. 1c). However, this did not result in a difference in the level of soil compaction when tall vs. short vegetation was compared when large grazers were present.

### Collembola community

**Fig. 2:**
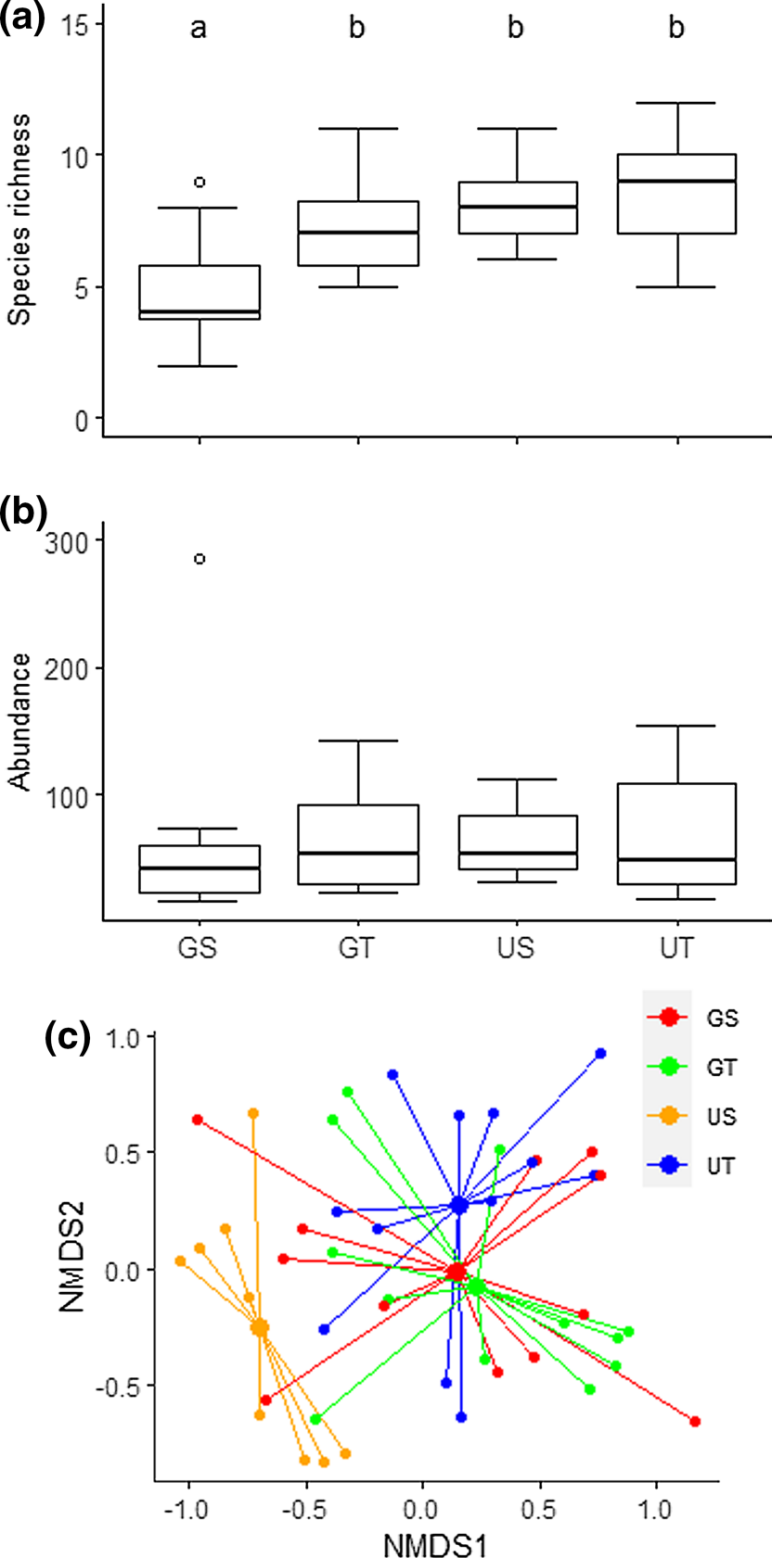
Effects of soil compaction and vegetation height on **a** species richness, **b** abundance (number of individuals per core) and **c** species composition of Collembola. The boxplots in figures **a** and **b** show range (whiskers), 25 and 75% quartiles (box), median (thick line) and outliers (circles), and figure **c** shows the NMDS ordination. In **a** letters indicate significant differences among treatments. Treatments: *GS* grazing, short vegetation, *GT* grazing, tall vegetation, *US* no grazing, short vegetation, *UT* no grazing, tall vegetation

We collected 2905 specimens comprising 19 species of 17 genera and 11 families. The three most abundant species were *Mesaphorura macrochaeta*, *Folsomia sexoculata* and *Isotoma anglicana* (see Online Resource 5). Species richness was lower in sites with soil compaction, but greater under tall vegetation (Table 1; Fig. 2a; Online Resource 5). When comparing the different treatments, specifically in short-grazed areas, a lower species richness was observed. Abundances of Collembola were not affected by grazing or vegetation height (Fig. 2b).

**Fig. 3:**
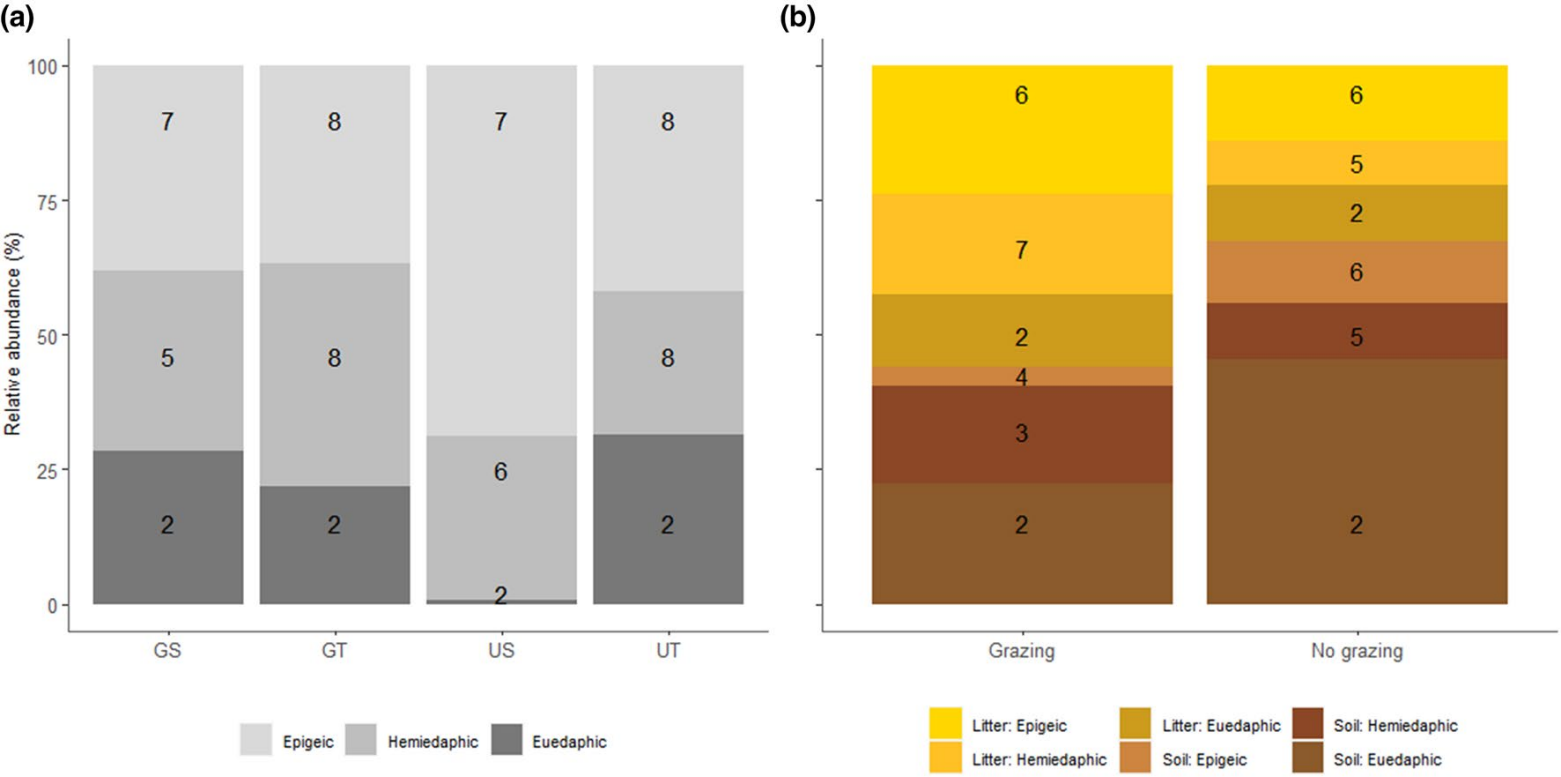
Relative abundance of the different life-forms of Collembola **a** within the soil across different treatments of soil compaction and vegetation height, and **b** each life-form separated in litter and soil layers in tall vegetation within grazed and ungrazed treatments. In **b** the top three layers represent the presence of life-form groups of Collembola in the litter layer (light colours) and the bottom three layers represent their presence in the soil layer (darker colours). The numbers in the bars indicate species richness within the life-form group of Collembola. Treatments: *GS* grazing, short vegetation, *GT* grazing, tall vegetation, *US* no grazing, short vegetation, *UT* no grazing, tall vegetation

When looking specifically at soil and litter in tall vegetation sites, we found in the grazed treatment a reduction from a total of 15 species in the litter layer (7.5 ± 0.92) to 9 species in the soil layer (4.3 ± 0.71), of which eight species were present in both layers (Fig. 3b, Online Resource 6). A total of 13 species were found in both layers of the ungrazed treatment (litter 6.8 ± 0.95; soil 6.3 ± 0.84), which included 11 similar species (Fig. 3b, Online Resource 6). However, we found no effect of stratum or grazing (or interaction) on Collembola species composition (PERMANOVA: *P* > 0.05 for all factors).

The species composition of the samples from the entire cores (non-separated layers) was significantly affected by grazer presence (PERMANOVA: *F*_1,44_ = 3.31, *P* < 0.01) and vegetation height (*F*_1,44_ = 2.48, *P* < 0.05), and their interaction (*F*_1,44_ = 2.72, *P* < 0.01), although the explanatory power of the model was relatively low (*R*^2^ = 0.17). The NMDS ordination showed a clear compositional separation of the ungrazed, short vegetation with the other three treatments (Fig. 2c). These differences were mostly driven by *Sminthurinus aureus*, *Parisotoma notabilis*, *F. sexoculata*, *I. anglicana* and *M. macrochaeta*, with particularly high abundances of the first two species and low abundance of *M. macrochaeta* in ungrazed, short vegetation (Online Resource 7).

### Collembola life-form

**Fig. 4:**
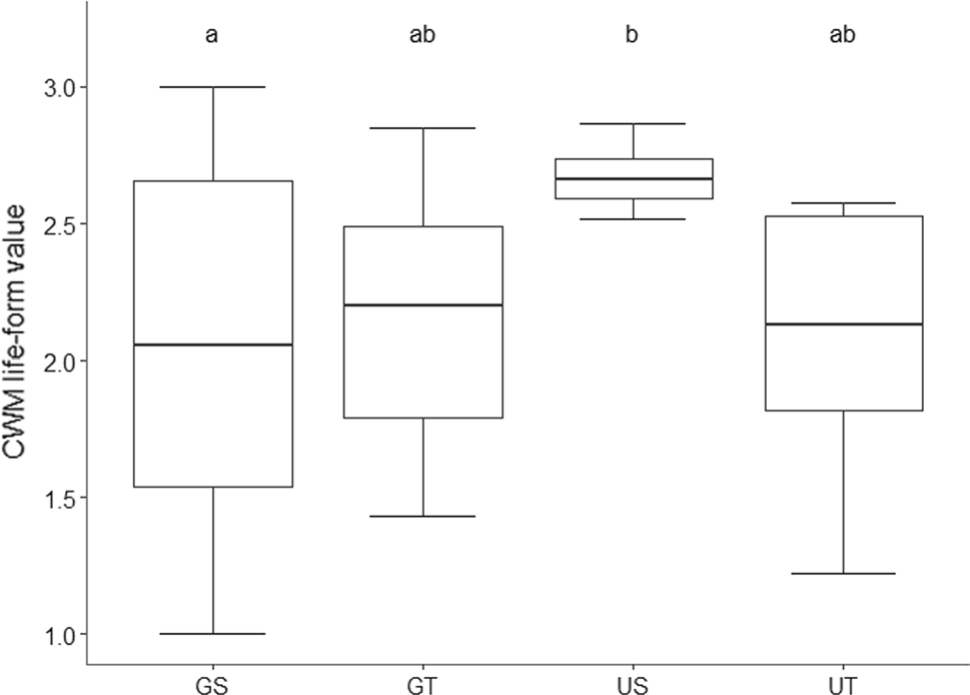
The effect of soil compaction and vegetation height on the community composition of Collembola expressed as community weighted mean (CWM) life-form values. Life-form value 1 indicates a pure euedaphic community, while value 3 indicates a pure epigeic community. Boxplots show range (whiskers), 25 and 75% quartiles (box), and median (thick line). Letters indicate significant differences among treatments. Treatments: *GS* grazing, short vegetation, *GT* grazing, tall vegetation, *US* no grazing, short vegetation, *UT* no grazing, tall vegetation

We found an interaction effect of soil compaction and vegetation height for CWM of life-form. CWM of life-form differed in short vegetation between grazed and ungrazed soils, with higher CWM values in the ungrazed treatment (Table 2; Fig. 4; Online Resource 2). This indicates that the abundance of epigeic species was greater in the ungrazed, short vegetation treatment than the grazed treatment (see also Fig. 3a).

**Table 2:** Significant effects of compaction and vegetation height on CWM life-form, epigeic, hemiedaphic and euedaphic species richness, abundances and species composition

Parameter	Collembola life-form	Compaction		Vegetation height		Compaction × vegetation height	
		Test statistic	*P*	Test statistic	*P*	Test statistic	*P*
CWM life-form	–					*F* = 4.53	< 0.05
Species richness	Epigeic	*W* = 355	< 0.05				
	Hemiedaphic	*W* = 348	< 0.05	*W* = 164.50	< 0.05		
	Euedaphic			*W* = 135.50	< 0.01		
Abundance (number of individuals per core)	Epigeic	*F* = 7.50	< 0.01				
	Hemiedaphic						
	Euedaphic			*F* = 11.45	< 0.01	*F* = 5.40	< 0.05
Species composition	Epigeic	*F* = 2.65	< 0.05				
	Hemiedaphic	*F* = 4.68	< 0.001	*F* = 2.62	< 0.05	*F* = 5.17	< 0.001
	Euedaphic			*F* = 8.57	< 0.001	*F* = 3.20	< 0.05

Species richness of epigeic and hemiedaphic species was lower with soil compaction. Both hemiedaphic and euedaphic species richness was greater with tall vegetation, but particularly with soil compaction for hemiedaphic species (Table 2; Online Resource 8, 9). Only the abundance of epigeic species was affected by soil compaction. This was specifically pronounced in short vegetation, with lower abundances with soil compaction compared with decompacted soil.

The abundance of hemiedaphic species was not affected by either compaction or vegetation height. The abundance of euedaphic species was greater in tall vegetation, particularly in decompacted soils (Table 2; Fig. 3a; Online Resource 9).

Epigeic and hemiedaphic species composition were significantly affected by soil compaction. Hemiedaphic and euedaphic species composition were affected by both vegetation height and the interaction with soil compaction (Table 2; Online Resource 9). The NMDS ordinations show some separation of ungrazed, short vegetation treatment with the grazed, short vegetation treatment for each life-form. In addition, hemiedaphic species also show compositional differences within decompacted soils between tall and short vegetation (Online Resource 9).

Separating soil and litter in tall vegetation sites we noticed the greater relative abundance of epigeic and hemiedaphic species in the litter layer of the grazed, soil compacted treatment compared with the ungrazed treatment, but contrastingly also the greater relative abundance of euedaphic species in the soil layer of the ungrazed treatment (Fig. 3b). The number of species of the different life-forms were relatively similar between the compaction treatments within each stratum (litter vs. soil; Fig. 3b).

## Discussion

EEs modulate their physical environment, each in their own way. When they co-occur in an ecological network their engineering effects can interact with each other and combined have a strong impact on the soil environment and its inhabitants. Here we show that the collective effect of large grazers, plants and bioturbating terrestrial amphipods indeed affect soil compaction, vegetation height and litter production, and ultimately cascade into Collembola communities. While these combined land-use and ecosystem engineering effects had an impact on Collembola species richness and life-form diversity, abundances were not affected contrary to our expectations. Below we discuss the combined impact of EEs on soil conditions and how shifts in soil porosity, vegetation height and litter layer thickness subsequently impact Collembola.

Consistent with other studies, all aspects of porosity we measured increased without grazers, which compact the soil through trampling (Schrama et al. 2013; Keshta et al. 2020). Soil porosity increased when grazers were excluded, particularly in presence of tall grass vegetation. Here, low light levels at the soil surface in combination with a thick litter layer attract *O. gammarellus,* which decompact the soil by their digging behaviour (Howison et al. 2015). While *O. gammarellus* is generally not found at grazed sites with a short vegetation, they do occur in tall vegetated patches in company of grazers (Schrama et al. 2012; Howison et al. 2015; Van Klink et al. 2015). Here, the environmental conditions, i.e. reduced light levels, thin litter layer for food and shelter and buffered climatic conditions, allow for their presence. However, this does not result in an increase in porosity as found under non-grazed conditions as the level of decompaction by *O. gammarellus* cannot keep up with the level of compaction by trampling large herbivores. In addition, the increase in porosity in the absence of large herbivores is mediated by the greater availability of litter within the soil column, as in the ungrazed tall vegetation treatment more afterlife material in the form of litter is generated (Bakker 1989; Widenfalk et al. 2015).

During inundation events, fresh sediment is deposited on top of litter, resulting in a layer consisting of alternating thin layers of sediment and buried litter on top of a soil column consisting of clay. This phenomenon is particularly pronounced in abandoned or ungrazed sites with tall vegetation, as vegetation structure (e.g. height, biomass) is an important factor in the process of sediment trapping (reviewed in Nolte et al. 2015), while litter itself also acts as a sediment trap (Rooth and Cornwell 2003). In this way, litter amplifies the effect of grazing release on habitable pore space and porosity also via the creation of favourable conditions for bioturbating amphipods. However, the comparable thickness of this layer of sediment-coated litter under short vegetation in the absence of grazing (Online Resource 1) and with very low abundances of *O. gammarellus* might suggest that porosity in the top layer is solely due to litter–sediment interactions. Despite the lower amount of litter in the ungrazed short vegetation treatment, there is sufficient decaying plant material present within the top layer attracting *O. gammarellus*. During the night *O. gammarellus* may visit these sites, when located in close proximity to tall vegetation patches (pers. obs. MPB), to feed on decaying plant material resulting in bioturbation of the top layer. This is corroborated by the intermediate level of air-filled porosity found in these ungrazed, short vegetation sites. Therefore, inferring the level of bioturbation from *O. gammarellus* densities when based on day-time sampling as in this study might underestimate their role as engineers in less favourable conditions (i.e. short vegetation, without grazers present).

Contrary to our expectation, the combined impact of EEs on soil compaction, as well as vegetation height, did not affect Collembola abundances. However, we did observe in tall vegetation sites that in the absence of grazing the larger part of the Collembola community (i.e. about 70%) was found in the soil layer in comparison with the litter layer, while in the grazed salt marsh this pattern was reversed. Collembola are known to respond strongly to litter availability, and litter is generally associated with greater abundances of Collembola (Potapov et al. 2020). Most Collembola are detritivores, thus litter provides nutritious resources. However, litter also offers habitable space, shelter and buffering against microclimate fluctuations (Takeda 1987; Fujii et al. 2020), thereby creating favourable habitat conditions for soil fauna, especially in compacted soils. Yet, while litter availability and thus food quantity and habitable space were greater in tall vegetation sites without grazers, relative abundances within the litter layer were not. In fact, here relative abundances were greater in the soil than in the litter layer. In addition, a severe drop in species number occurred from the litter to soil layer (from 15 to 9 species, respectively) in the grazed treatment, while the total number of species in each stratum was similar (13 species each) in the ungrazed treatment. The more favourable conditions in soil versus litter can be explained by a combination of an increase in soil porosity throughout the soil column and the availability of food in the top layer of the soil in the ungrazed sites, the latter due to greater quantities of buried litter due to sedimentation and bioturbation of *O. gammarellus*. The high amount of buried litter may combine the positive effect on habitable space and food quantity with the climate buffering effects of the soil on soil fauna. As Collembola are known to be sensitive to drought (Lindberg and Bengtsson 2006; Makkonen et al. 2011) living and feeding below the litter layer away from the more dry conditions on the soil surface may explain the observed relative abundance patterns in soil vs litter as a function of soil compaction by large herbivores. On the grazed salt marsh presence of a litter layer may compensate for a low soil porosity, while at the ungrazed salt marsh, litter lower down the soil column positively affects relative abundances and species richness through the provisioning of both food and habitat for Collembola. However, following this line of reasoning one would expect not only a shift in the relative abundance of Collembola between litter and soil, but also a significantly higher abundance in the absence of grazing, especially under tall vegetation. Here, the presence of the thickest litter layer, a thick layer of buried litter and a high soil porosity should result in the most optimal conditions for Collembola. The only explanation we can think of is that under these conditions soil temperature is probably the lowest of all treatments, which might impact population growth of Collembola.

The combination of soil compaction, short vegetation and low litter availability did affect species richness of Collembola, specifically of hemiedaphic and euedaphic species. This is to be expected as these are litter and deep-soil dwellers and thus most affected when smaller pore spaces become even more reduced and food availability is impaired due to less available litter as biomass is ingested by herbivores (Heisler and Kaiser 1995; Larsen et al. 2004). However, most striking was the low abundance of euedaphic species in the ungrazed, short vegetation treatment, which explains the observed differences in Collembola community composition between this treatment and the other three treatments in which the species compositions were all similar to each other. This was attributed to a severe drop of *Mesaphorura macrochaeta* compared with the other treatments (see species abundances in Online Resource 5). This species has been found to decline in soils with greater bulk densities (Larsen et al. 2004), indicating that even this small euedaphic species responds to soil compaction, but this was not observed here. One possible explanation could be a higher predation pressure in the short vegetation without grazers. Predation, for example by predatory mites reduces Collembola abundances, and is a trophic driver of community dynamics. Cortet et al. (2003) found a decrease in *M. macrochaeta* in combination with the predaceous mite *Hypoaspis aculeifer*. Predaceous mites can be very abundant on salt marshes (Haynert et al. 2017; Schrama et al. 2017) and were recorded from our soil cores, but not identified to species. Indeed, the plots of the ungrazed, short vegetation treatment often included high abundances of mites (data not shown) with concomitant generally low abundances of *M. macrochaeta*. Conversely, the plot with the lowest number of mites was the plot with the highest abundance of *M. macrochaeta*. While this relatively high number of specimens was recorded in only one core, and *M. macrochaeta* specifically is known to aggregate (Niklasson et al. 2000), it could very well be that the individuals *M. macrochaeta* aggregated to these safer conditions, explaining the high abundance found in one of the soil cores. However, what drives the distribution of mites remains to be determined.

In short, structurally engineered abiotic state changes generate biotic effects within ecological networks. Grazing, trampling herbivores, vegetation structure and bioturbating soil fauna modify the physical environment of Collembola particularly through changes in soil porosity and litter layer thickness. These changes do not so much change overall abundances and richness, however it does change functional community composition as the relative abundance of Collembola life-forms is affected within different soil strata (Berg et al. 1998; Berg and Bengtsson 2007; Krab et al. 2010). These changes in relative abundance of functional groups may affect ecosystem functioning as processes such as decomposition, and thus soil quality (Faber 1991; Hopkin 2007; Berg et al. 2001) within these different layers become modified, with potential cascading effects on other trophic levels. We expect this to be applicable to other microarthropod groups. Ecosystem engineering networks do not only directly affect ecosystem functioning, but also indirectly via soil fauna community composition.

## Supplementary Information

Below is the link to the electronic supplementary material.

Supplementary file1 (PDF 441 KB)

## Data Availability

The datasets used and/or analysed during the current study are available from the corresponding author on reasonable request.
