# Supplementary material for: Impact of three co-occurring physical ecosystem engineers on soil Collembola communities
Source: Oecologia. 2022 Apr 7;198(4):1085–96. doi: 10.1007/s00442-022-05152-5 (PMC9056452; doi:10.1007/s00442-022-05152-5)
Supplement: Supplementary file 1 — Supplementary file1 (PDF 441 KB) [file 442_2022_5152_MOESM1_ESM.pdf]

Electronic Supplemental Material

Impact of three co-occurring physical ecosystem engineers on soil Collembola communities

D.D.G Lagendijk[1,2,*], D. Cueva-Arias[1], A.R. Van Oosten[1] & M.P. Berg[1,3]

[1]Department of Ecological Science – Section Animal Ecology, Vrije Universiteit Amsterdam, De Boelelaan 1085, 1081 HV Amsterdam, The Netherlands

[2]School of Life Sciences, University of KwaZulu-Natal, Private Bag X01, Scottsville, 3209, South Africa

[3]Conservation and Community Ecology Group, Groningen Institute for Evolutionary Life Sciences, University of Groningen, P.O. Box 11103, 9700 CC Groningen, The Netherlands

[*] georgette.lagendijk@gmail.com

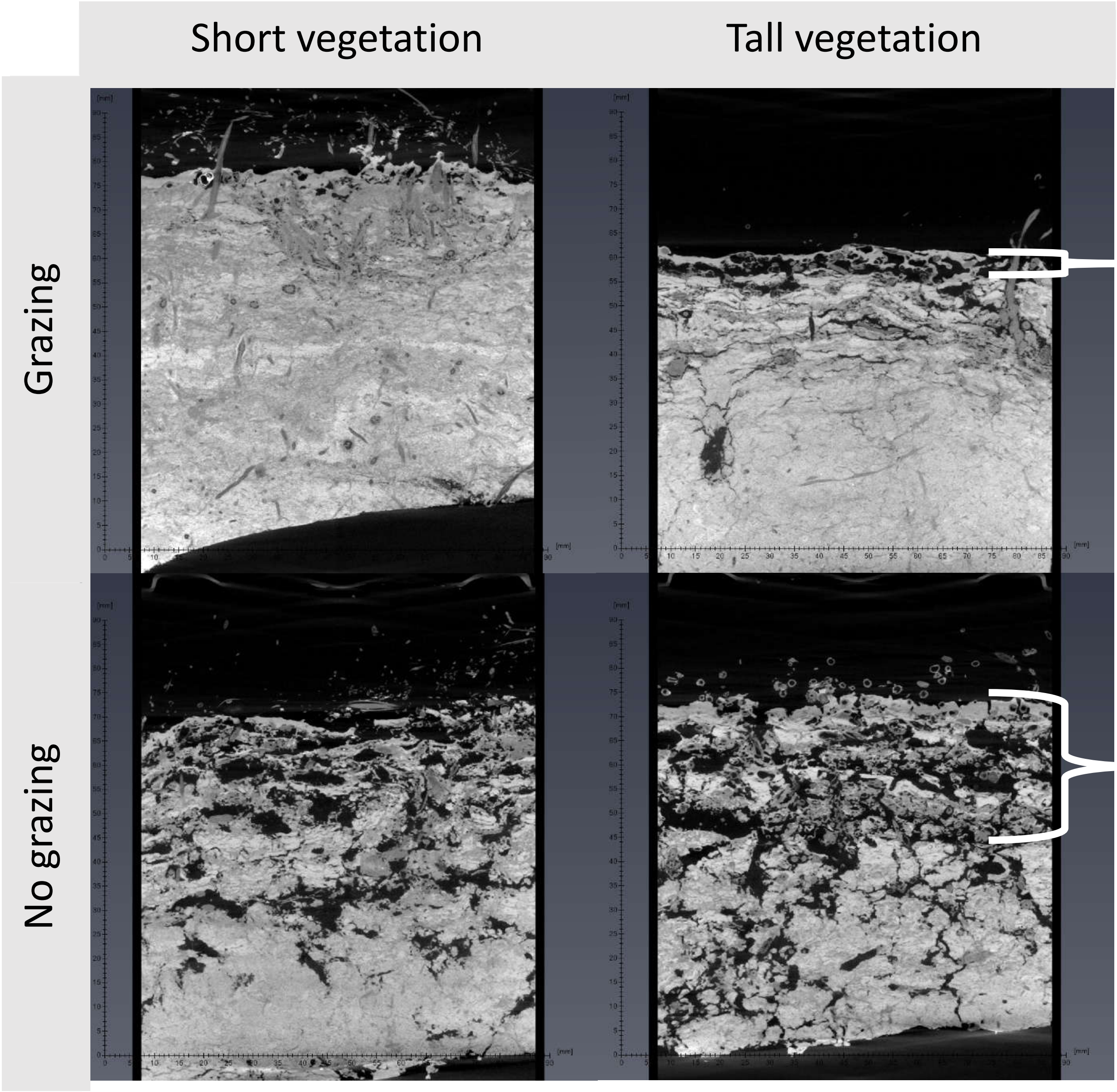

Online Resource 1.

X-ray tomography images of cross sections of soil columns (about 7 cm height) of the four different treatments, showing the effect of grazing (vertical panels; soil compaction) and vegetation height (horizontal panels) on soil structure. The ungrazed soils show more pores (shown as black), while the grazed soils show more compaction (shown as light grey). The white braces in the tall vegetation treatments indicate a layer of sediment mixed with litter on top of a clay layer.

Online Resource 2.

Means ± SE of ecosystem engineering variables, soil properties and Collembola species richness and abundances at the salt marsh of the barrier island of Schiermonnikoog, The Netherlands.

Variable	Grazing, short vegetation	Grazing, tall vegetation	Ungrazed, short vegetation	Ungrazed, tall vegetation
Vegetation height (cm)	2.33 ± 0.29	27.05 ± 1.82	5.87 ± 0.46	40.20 ± 2.31
Litter layer thickness (cm)	0.63 ± 0.04	1.36 ± 0.14	0.77 ± 0.10	2.43 ± 0.15
Orchestia abundance	2.86 ± 2.05	45.71 ± 11.59	1.43 ± 0.78	60.36 ± 17.26
Air-filled porosity (%)	1.29 ± 0.15	2.39 ± 0.22	11.19 ± 0.50	17.64 ± 2.12
Number of pores	10403 ± 807	14296 ± 950	19739 ± 2022	23895 ± 1092
CWM* life-form	2.10 ± 0.19	2.15 ± 0.14	2.68 ± 0.04	2.10 ± 0.13
Total Collembola species richness	5.00 ± 0.69	7.25 ± 0.54	8.00 ± 0.53	8.58 ± 0.65
Total Collembola abundance (number of individuals per core)	61.58 ± 21.13	66.00 ± 11.71	62.77 ± 9.24	67.50 ± 13.40

* Community weighted mean

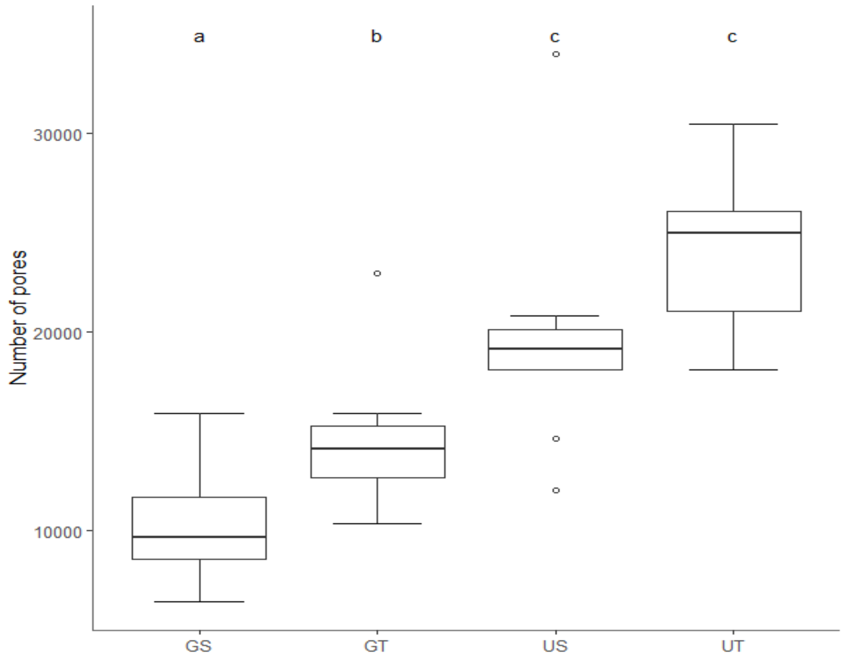

Online Resource 3.

Effect of grazing (soil compaction) and vegetation height on the number of soil pores. Letters indicate significant differences among treatments. Treatments: GS: grazing, short vegetation; GT: grazing, tall vegetation; US: no grazing, short vegetation; UT: no grazing, tall vegetation.

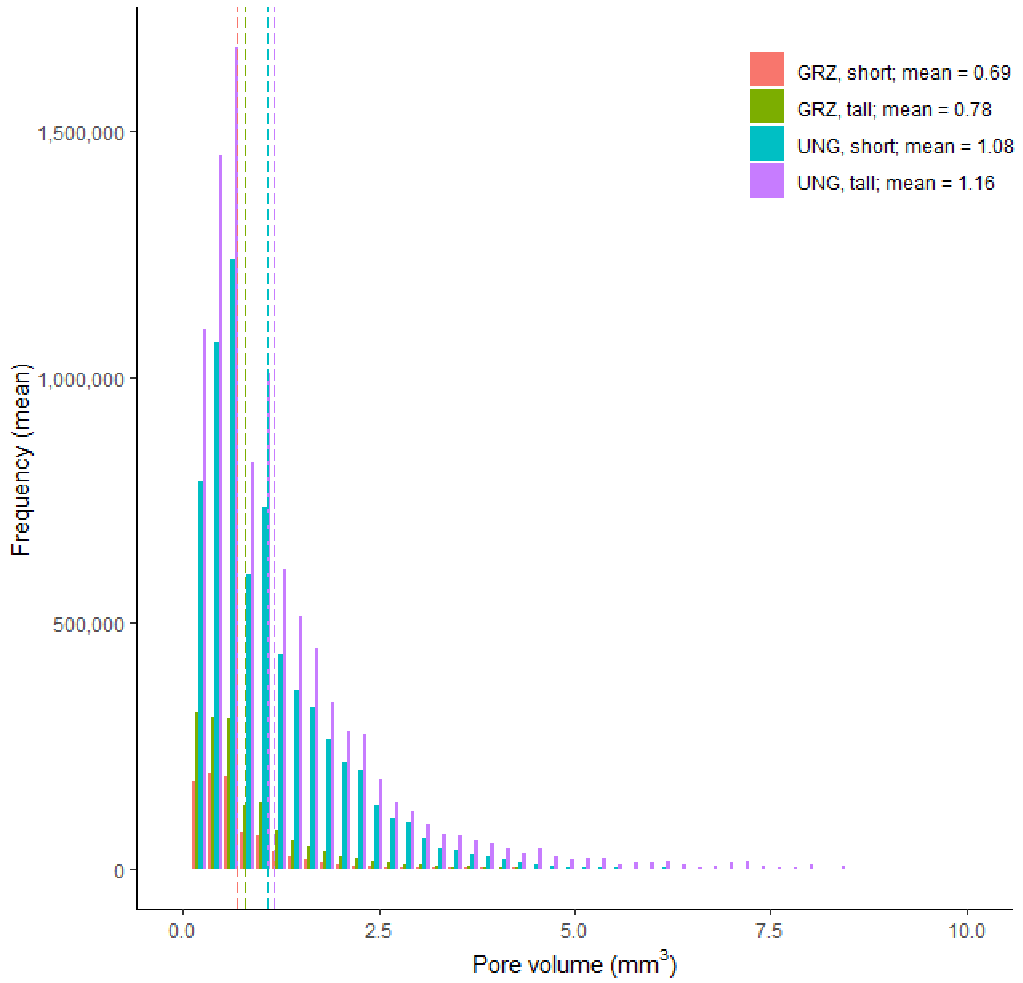

Online Resource 4.

Effect of grazing (soil compaction) and vegetation height on the mean (n=4) soil pore space frequency distribution. Treatments: GS: grazing, short vegetation; GT: grazing, tall vegetation; US: no grazing, short vegetation; UT: no grazing, tall vegetation. The dashed lines indicate the average pore volume in each of the four treatments with the different colours referring to the treatments.

Online Resource 5.

Collembola species encountered in four treatments* (level of soil compaction and difference in vegetation height) on the salt marsh of the barrier island of Schiermonnikoog, The Netherlands, in 2017. Greatest mean abundance (number of individuals per core) per treatment in bold.

Species	Life-form**	GS	GT	US	UT
Dicyrtomina minuta	Epi	0.58 ± 0.31	4.58 ± 3.01	3.56 ± 1.68	3.33 ± 1.14
Isotoma anglicana	Epi	5.80 ± 2.70	2.40 ± 1.10	6.00 ± 2.20	8.80 ± 5.70
Isotoma riparia	Epi	1.50 ± 1.10	0.92 ± 0.4	0.22 ± 0.15	0.92 ± 0.45
Lepidocyrtus cyaneus	Epi	0.75 ± 0.28	1.00 ± 0.46	1.33 ± 0.58	8.08 ± 3.79
Sminthurinus aureus	Epi	4.50 ± 1.80	3.70 ± 2.00	**19.2 ± 6.1**	5.30 ± 2.50
Sminthurus nigromaculatus	Epi	2.80 ± 1.00	2.80 ± 1.20	9.40 ± 2.60	3.20 ± 1.00
Sminthurus viridis	Epi	0	0.08 ± 0.08	0	0.17 ± 0.17
Sphaeridia pumilis	Epi	0.08 ± 0.08	0.33 ± 0.26	5.78 ± 2.28	2.42 ± 1.62
Brachystomella parvula	Hemi	0.42 ± 0.34	1.50 ± 1.00	0.33 ± 0.24	0.17 ± 0.17
Ceratophysella succinea	Hemi	0	0.17 ± 0.17	0	0.50 ± 0.42
Folsomia sexoculata	Hemi	7.67 ± 4.8	**23.3 ± 8.19**	4.44 ± 1.84	1.25 ± 0.52
Friesea truncata	Hemi	4.92 ± 1.26	5.42 ± 1.14	1.33 ± 0.67	4.58 ± 0.73
Halisotoma maritima	Hemi	0	0.17 ± 0.17	0.78 ± 0.28	3.92 ± 1.57
Hypogastrura viatica	Hemi	0.08 ± 0.08	0.58 ± 0.36	1.78 ± 1.35	2.42 ± 1.06
Parisotoma notabilis	Hemi	0.17 ± 0.17	0.25 ± 0.25	7.78 ± 2.13	0.58 ± 0.36
Pseudosinella alba	Hemi	0	0	0	0.08 ± 0.08
Sminthurides malmgreni	Hemi	0	0.08 ± 0.08	0	0
Mesaphorura macrochaeta	Eu	**31.7 ± 23.0**	13.1 ± 5.38	0.56 ± 0.24	**20.0 ± 7.25**
Thalassaphorura debilis	Eu	0.67 ± 0.67	5.58 ± 2.86	0.11 ± 0.11	1.83 ± 0.91

[*]GS: grazing, short vegetation; GT: grazing, tall vegetation; US: no grazing, short vegetation; UT: no grazing, tall vegetation.

[**]Life-form: Epi: epigeic; Eu: euedaphic; Hemi: hemiedaphic.

Online Resource 6.

Collembola species encountered in the litter and soil strata in grazed (GRZ) and ungrazed (UNG) treatments on the salt marsh of the barrier island of Schiermonnikoog, The Netherlands, in 2017. Greatest mean abundance (number of individuals per core) per treatment in bold.

Species	Life-form*	GRZ, litter	GRZ, soil	UNG, litter	UNG, soil
Entomobrya lanuginosa	Atmo	0	1.00 ± 0.82	0	0
Dicyrtomina minuta	Epi	4.33 ± 2.08	0	4.00 ± 1.63	2.00 ± 1.48
Isotoma anglicana	Epi	12.3 ± 7.27	2.00 ± 1.48	5.17 ± 2.34	10.3 ± 5.45
Isotoma riparia	Epi	0.67 ± 0.49	0	0	0
Lepidocyrtus cyaneus	Epi	7.67 ± 5.04	0.17 ± 0.17	5.00 ± 2.50	1.50 ± 0.72
Sminthurinus aureus	Epi	1.33 ± 1.33	0.17 ± 0.17	0.83 ± 0.48	1.83 ± 1.64
Sminthurus nigromaculatus	Epi	1.17 ± 0.31	0	1.17 ± 0.48	0.50 ± 0.34
Sminthurus viridis	Epi	0	0	1.00 ± 0.37	0
Sphaeridia pumilis	Epi	0	0	0	0.33 ± 0.33
Brachystomella parvula	Hemi	0.17 ± 0.17	0	0.17 ± 0.17	0
Ceratophysella succinea	Hemi	0.83 ± 0.83	0	0	0
Folsomia sexoculata	Hemi	12.7 ± 4.35	13.3 ± 9.23	2.00 ± 2.00	4.67 ± 2.55
Friesea truncata	Hemi	2.83 ± 1.01	9.50 ± 3.94	2.33 ± 1.12	5.00 ± 2.07
Halisotoma maritima	Hemi	0	0	0	0.33 ± 0.33
Hypogastrura viatica	Hemi	3.17 ± 2.12	1.50 ± 0.81	6.17 ± 2.50	4.00 ± 2.41
Megalothorax minimus	Hemi	0.17 ± 0.17	0	0.33 ± 0.33	0.67 ± 0.49
Sminthurides malmgreni	Hemi	0.50 ± 0.50	0	0	0
Mesaphorura macrochaeta	Eu	**49.5 ± 43.1**	**43.8 ± 26.2**	**16.8 ± 6.80**	**103.0 ± 47.5**
Thalassaphorura debilis	Eu	0.67 ± 0.49	7.33 ± 4.67	0.67 ± 0.67	1.67 ± 1.67

*Life-form: Atmo: atmobiotic; Epi: epigeic; Eu: euedaphic; Hemi: hemiedaphic.

Online Resource 7.

SIMPER analyses of the Collembola species composition among treatments on the salt marsh of the barrier island of Schiermonnikoog, The Netherlands, in 2017.

Treatments (% dissimilarity)	Species	Average abundance		Average dissimilarity %	Cumulative %
		GRZ, short	GRZ, tall		
GRZ, short vs. GRZ, tall (73.7%)	*Folsomia sexoculata*	7.67	23.3	19.2	26.1
	Mesaphorura macrochaeta	31.7	13.1	17.8	50.3
	Isotoma anglicana	5.83	2.42	7.13	60.0
	Sminthurinus aureus	4.50	3.67	6.54	68.9
	Friesea truncata	4.92	5.42	4.55	75.1
	Dicyrtomina minuta	0.58	4.58	4.08	80.6
	Sminthurus nigromaculatus	2.75	2.83	3.78	85.7
	Thalassaphorura debilis	0.67	5.58	3.64	90.7
	Isotoma riparia	1.50	0.92	1.95	93.3
	Lepidocyrtus cyaneus	0.75	1.00	1.49	95.3
	Brachystomella parvula	0.42	1.50	1.42	97.3
	Hypogastrura viatica	0.08	0.58	0.78	98.3
	Sphaeridia pumilis	0.08	0.33	0.43	98.9
	Parisotoma notabilis	0.17	0.25	0.25	99.3
	Ceratophysella succinea	0	0.17	0.20	99.5
	Sminthurus viridis	0	0.08	0.13	99.7
	Sminthurides malmgreni	0	0.08	0.12	99.9
	Halisotoma maritima	0	0.17	0.10	100
	Pseudosinella alba	0	0	0	100

Treatments (% dissimilarity)	Species	Average abundance		Average dissimilarity %	Cumulative %
		UNG, short	UNG, tall		
UNG, short vs. UNG, tall (79.1%)	*Sminthurinus aureus*	19.2	5.33	13.5	17.0
	Mesaphorura macrochaeta	0.56	20.0	12.3	32.6
	Isotoma anglicana	6.00	8.75	8.15	42.9
	Parisotoma notabilis	7.78	0.58	7.30	52.1
	Sminthurus nigromaculatus	9.44	3.17	6.28	60.0
	Lepidocyrtus cyaneus	1.33	8.08	6.17	67.8
	Sphaeridia pumilis	5.78	2.42	4.64	73.7
	Dicyrtomina minuta	3.56	3.33	3.74	78.4
	Folsomia sexoculata	4.44	1.25	3.66	83.1
	Friesea truncate	1.33	4.58	3.43	87.4
	Halisotoma maritima	0.78	3.92	3.39	91.7
	Hypogastrura viatica	1.78	2.42	2.44	94.7
	Thalassaphorura debilis	0.11	1.83	2.28	97.6
	Isotoma riparia	0.22	0.92	0.69	98.5
	Ceratophysella succinea	0	0.50	0.57	99.2
	Brachystomella parvula	0.33	0.17	0.47	99.8
	Sminthurus viridis	0	0.17	0.10	99.9
	Pseudosinella alba	0	0.08	0.05	100
	Sminthurides malmgreni	0	0	0	100

Treatments (% dissimilarity)	Species	Average abundance		Average dissimilarity %	Cumulative %
		GRZ, short	UNG, short		
GRZ, short vs. UNG, short (80.7%)	*Mesaphorura macrochaeta*	31.7	0.56	14.3	17.8
	Sminthurinus aureus	4.50	19.2	14.3	35.5
	Folsomia sexoculata	7.67	4.44	9.14	46.9
	Parisotoma notabilis	0.17	7.78	8.34	57.2
	Isotoma anglicana	5.83	6.00	8.26	67.4
	Sminthurus nigromaculatus	2.75	9.44	6.86	75.9
	Friesea truncata	4.92	1.33	4.99	82.1
	Sphaeridia pumilis	0.08	5.78	4.73	88.0
	Dicyrtomina minuta	0.58	3.56	3.57	92.4
	Lepidocyrtus cyaneus	0.75	1.33	1.47	94.2
	Hypogastrura viatica	0.08	1.78	1.42	96.0
	Isotoma riparia	1.50	0.22	1.35	97.7
	Halisotoma maritima	0	0.78	0.89	98.8
	Brachystomella parvula	0.42	0.33	0.73	99.7
	Thalassaphorura debilis	0.67	0.11	0.26	100
	Ceratophysella succinea	0	0	0	100
	Sminthurides malmgreni	0	0	0	100
	Sminthurus viridis	0	0	0	100
	Pseudosinella alba	0	0	0	100

Treatments (% dissimilarity)	Species	Average abundance		Average dissimilarity %	Cumulative %
		GRZ, short	UNG, short		
GRZ, tall vs. UNG, tall (74.1%)	*Folsomia sexoculata*	23.3	1.25	15.4	20.8
	Mesaphorura macrochaeta	13.1	20.0	13.1	38.4
	Isotoma anglicana	2.42	8.75	6.74	47.5
	Lepidocyrtus cyaneus	1.00	8.08	6.34	56.1
	Sminthurinus aureus	3.67	5.33	5.67	63.7
	Thalassaphorura debilis	5.58	1.83	4.98	70.4
	Dicyrtomina minuta	4.58	3.33	4.35	76.3
	Halisotoma maritima	0.17	3.92	3.66	81.2
	Sminthurus nigromaculatus	2.83	3.17	3.43	85.8
	Friesea truncata	5.42	4.58	3.01	89.9
	Hypogastrura viatica	0.58	2.42	1.91	92.5
	Sphaeridia pumilis	0.33	2.42	1.53	94.6
	Isotoma riparia	0.92	0.92	1.18	96.2
	Brachystomella parvula	1.50	0.17	1.14	97.7
	Ceratophysella succinea	0.17	0.50	0.72	98.7
	Parisotoma notabilis	0.25	0.58	0.62	99.5
	Sminthurus viridis	0.08	0.17	0.20	99.8
	Sminthurides malmgreni	0.08	0	0.10	99.9
	Pseudosinella alba	0	0.08	0.05	100

Online Resource 8.

Species richness and abundances (mean ± SE) of epigeic, hemiedaphic and euedaphic Collembola species on the salt marsh of the barrier island of Schiermonnikoog, The Netherlands.

Variable	Life-form	Grazing, short vegetation	Grazing, tall vegetation	Ungrazed, short vegetation	Ungrazed, tall vegetation
Species richness	Epigeic	2.75 ± 0.65	3.25 ± 0.45	4.56 ± 0.38	4.25 ± 0.65
	Hemiedaphic	1.50 ± 0.34	2.75 ± 0.33	2.89 ± 0.48	3.08 ± 0.26
	Euedaphic	0.75 ± 0.18	1.25 ± 0.18	0.56 ± 0.24	1.25 ± 0.13
Abundance	Epigeic	16.00 ± 5.46	15.83 ± 4.34	45.56 ± 9.15	32.17 ± 8.34
(number of individuals per core)	Hemiedaphic	13.25 ± 4.97	31.50 ± 8.51	16.44 ± 2.46	13.50 ± 2.36
	Euedaphic	32.33 ± 23.60	18.67 ± 7.50	0.67 ± 0.33	21.83 ± 6.96

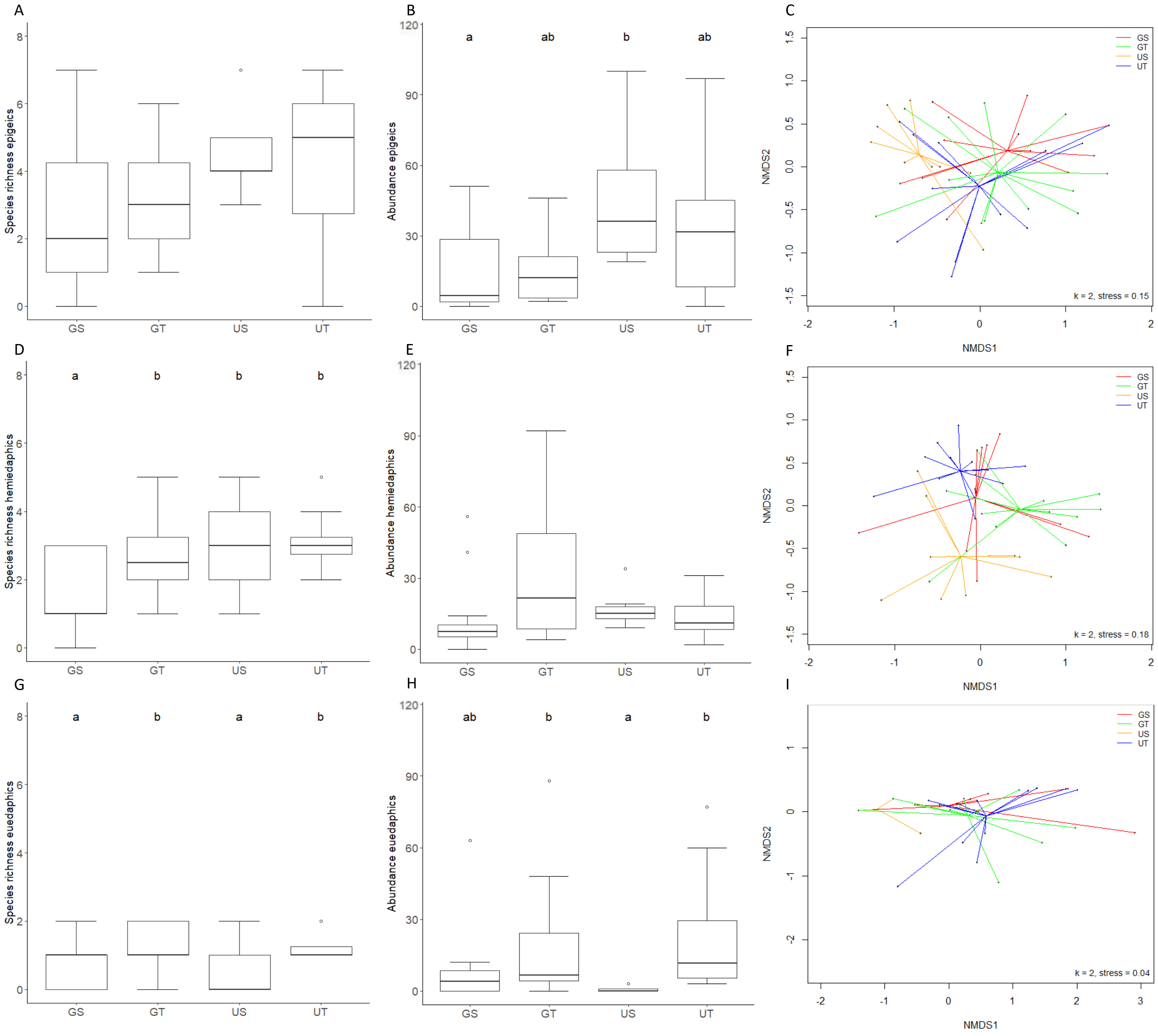
A
Species richness epigeics
GS
GT
US
UT
B
Abundance epigeics
a
ab
b
ab
C
NMDS1
NMDS2
k = 2, stress = 0.15
D
Species richness hemiedaphics
a
b
b
b
E
Abundance hemiedaphics
F
k = 2, stress = 0.18
G
Species richness euedaphics
a
b
a
b
H
Abundance euedaphics
ab
b
a
b
I
k = 2, stress = 0.04

Online Resource 9.

Effects of soil compaction and vegetation height on species richness (A,D,G), abundance (number of individuals per core; B,E,H) and species composition (C,F,I) of epigeic, hemiedaphic and euedaphic Collembola, respectively. Letters indicate significant differences among treatments. Treatments: GS: grazing, short vegetation; GT: grazing, tall vegetation; US: no grazing, short vegetation; UT: no grazing, tall vegetation.
